# Convergence of alimentary air inflation and adult non-feeding in insects, and possible adaptive functions

**DOI:** 10.1371/journal.pone.0351543

**Published:** 2026-06-11

**Authors:** Hollister W. Herhold, Steven R. Davis, Rebecca Jean A. Millena, Anna Eichert, Amanda Markee, David A. Grimaldi

**Affiliations:** 1 American Museum of Natural History, Division of Invertebrate Zoology, New York, United States of America; 2 School of Life Science and Technology, Institute of Science Tokyo, Tokyo, Japan; 3 University of Rochester, Rochester, New York, United States of America; 4 American Museum of Natural History, Richard Gilder Graduate School, New York, United States of America; University of Zagreb Faculty of Teacher Education, CROATIA

## Abstract

Among the many diverse traits of insects, the most speciose and successful terrestrial animals, is an incredible range of lifespans. While some are quite long-lived, such as cicadas (years as larvae) or termite queens (decades as adults), many insects, such as mayflies (Insecta: Ephemeroptera), have exceedingly short adult lifespans that serve essentially just for mating and selecting oviposition sites, foregoing feeding to reproduce as quickly as possible. This behavior is correlated with mouthparts that are highly reduced or even absent, and the alimentary system is converted into an air-filled space, possibly non-functional for digestion. The order-wide phenomenon of the co-opted gut is found in only one other group, the twisted-wing parasites (Insecta: Strepsiptera). Here, we present micro-CT scans and volume measurements (body and alimentary air) that reveal a previously undocumented inflated alimentary system in non-feeding species from five additional insect orders: Plecoptera, Embioptera, Megaloptera, Lepidoptera, and Diptera. The association between reduction or complete loss of adult feeding and a large volume of air in the gut is statistically highly significant. The reduction of mouthparts in these taxa reflects non-feeding in adults, indicating that convergence of this trait with alimentary inflation probably has adaptive functions. We discuss several, nonexclusive ways in which an inflated alimentary system can be adaptive for adult insects.

## Introduction

Insects are some of the most successful organisms, able to adapt and thrive in nearly every habitat on Earth. Insects display an incredible diversity of form and size, from tiny midges and parasitoid wasps less than 1 mm in wingspan to Hercules beetles the size of a human hand, and insect lifespans are equally wide-ranging. Migrating monarch butterflies live several months, queen honeybees may survive two or three years, and some termite queens may thrive for decades. The non-mating immature stage (larvae/nymphs) of many insects is usually longer than the reproductive adult stage, sometimes substantially so, such as the 13-year periodical cicadas of the genus *Magicicada*. Some insects with the shortest adult lifespans, several days or in some cases just a few hours, are so constrained to mate that they do not feed and rely entirely on energy stores accumulated as larvae.

The approximately 3,000 species of the insect order Ephemeroptera, known as mayflies, have some of the shortest adult lifespans. Adult mayflies persist for a few days, sometimes only several hours, forgoing any feeding in their quest to reproduce as quickly as possible. The adult is completely reliant on energy reserves gained during the aquatic immature larval stage, which can last many months [1]. All mayflies exhibit adult non-feeding and relatively short lifespans. This behavior correlates with observable physical changes as well, as many adult mayflies possess reduced mouthparts, with some even completely absent in numerous species [2].

Strepsiptera is the only other insect order where adult non-feeding is present in all (approximately) 600 species. Strepsiptera males do not feed as adults and have exceedingly short lifespans, typically less than a day (in some species, only a few hours) spent flying from host to host in search of females [3,4]. The flightless endoparasitic strepsipteran females spend their entire lives surviving in their host, awaiting the arrival of males (with the notable exception of free-living females in the family Mengenillidae [5]). Interestingly, adult male Strepsiptera also have highly reduced mouthparts, like mayflies. Female adults also feature reduced, vestigial mouthparts on their cephalothorax, yet they do not take in food as mature adults, having ceased absorbing nutrients from the host after completing development [6].

All Saturniidae (silk moths, Lepidoptera, ~ 3,200 species) also exhibit short adult lifespans, along with the absence or reduced development of mouthparts [7]. Larvae feed and accumulate extensive fat reserves for several weeks — energy storage that is used by non-feeding adults for reproduction and dispersal. As adults, many silk moths live between one and two weeks, relying solely on these fat reserves [8]. Other lepidopteran groups, including some species of Sphingidae (hawkmoths, ~ 1,450 species) have also lost the ability to feed as adults, with the consequent reduction of mouthparts [9]. This life history trait is not present amongst all members of Lepidoptera, however, as adult feeding is highly variable across groups. Indeed, the great proportion of adult Lepidoptera have well developed mouthparts used for feeding on nectar, plant exudates, and/or sodium-rich fluids.

Non-feeding adult insects with short lifespans are found sporadically in other orders. Several species of Plecoptera (stoneflies) are suspected to not feed as adults, although records are inconclusive [10]. A few Diptera (flies) have a short adult lifespan and do not feed, such as species of hover flies in the genus *Microdon* (Diptera: Syrphidae) [11]. *Bombyx mori* (Lepidoptera: Bombycidae) silkworm caterpillars are strictly monophagous on mulberry leaves [12–14]; the adults do not feed. But most insect orders have a wide range of species that feed both during immature and reproductive adult stages, as well as insects that do not feed as adults. Only in Ephemeroptera and Strepsiptera is this adult non-feeding trait present throughout the entire order.

In addition to reduced mouthparts, it has been known that mayfly adults of both sexes [15–18] and male Strepsiptera [19,20] possess an additional, internal modification associated with the reduced feeding apparatus: the alimentary canal is inflated with air and may be non-functional for digestion. This balloon-like modification of the alimentary canal in Ephemeroptera and Strepsiptera has not previously been noted in any other insect order.

While conducting a broad comparative study of insect respiratory architectures using computed tomography (micro-CT scanning) [17], insects from five additional orders were found to have an inflated and likely non-functional digestive tract. While several of these insects are already known to not feed, reduced or absent mouthparts observed in the others suggests a newly documented non-feeding adult stage. This pattern indicates that an inflated alimentary canal is associated with non-feeding adults and may therefore represent an adaptive trait (e.g., exaptation [21]). Using micro-CT, a non-destructive technique that enables detailed examination of fine anatomical structures *in situ* [22], here we investigate the association of non-feeding behaviors, modified or reduced mouthparts, and modified digestive morphology amongst seven insect orders.

## Methods

All specimens were collected live. Mayflies and the *Bombyx mori* specimen were obtained from live cultures of the Division of Invertebrate Zoology of the American Museum of Natural History (AMNH, New York, NY). All other specimens were collected in the New York/New Jersey Metropolitan Area and the Black Rock Forest (Cornwall, NY).

Specimens were killed as soon as possible after capture by freezing to −80℃ (see [17] for detailed discussion of preservation protocols). Pinned museum specimens are unsuitable for determining internal air spaces as tissues desiccate and distort over time. Likewise, specimens stored in alcohol were unusable as fluid infilling makes determination of air spaces difficult if not impossible. All specimens were thawed to room temperature prior to scanning.

Micro-CT scanning was performed at the AMNH Microscopy and Imaging Facility using a GE Phoenix v|tome|x s240 Micro-CT Scanner equipped with a 180 kV X-ray emitter and a DXR-250 detector (refer to Table 1 for scanning parameters). Volume reconstruction was performed using datos|x 2.3.2. Post-processing was done using methods detailed in [17]. Segmentations were performed using 3D Slicer versions 4.1 through 5.6 [23], and visualizations were rendered in Blender [24]. Figures were composed in Adobe Illustrator.

**Table 1:** Micro-CT scanning parameters.

Order	Taxon	Voxel size (mm)	Voltage (kV)	Current (µA)	Averaged frames	Frame skip	Exposure (msec)
Ephemeroptera	*Ephemera* sp. ♀	0.0131	60	200	2	1	200
Ephemeroptera	*Neocloeon triangulifer* (subimago) ♀	0.0041	80	250	4	1	400
Ephemeroptera	*Neocloeon triangulifer* ♀	0.0023	90	170	4	1	400
Plecoptera	*Isoperla* sp. ♀	0.0060	60	400	3	1	333
Embioptera	*Oligotoma nigra* ♂	0.0053	90	180	3	0	400
Megaloptera	*Corydalus cornutus* ♀	0.0189	70	400	3	1	333
Strepsiptera	*Xenos peckii*. ♂	0.0036	100	150	3	1	750
Lepidoptera	*Bombyx mori* (domestic) ♀	0.0113	70	285	4	1	333
Diptera	*Ogcodes* sp. ♂	0.0072	90	300	4	1	400

All specimens are adults except the *Neocloeon triangulifer* subimago.

Development of external and internal appendages and other elements of the mouthparts, and associated structures, were scored by dissection of the head. The head was removed, soaked in warm 10% KOH for several hours to macerate the muscles and other tissues, provide some clarity to the sclerotized portions, and examine internal structures (e.g., lacinia, cibarium, tentorium, etc). The head and mouthparts were then rinsed in water, then 1:1 water:70% ethanol, then 70% ethanol, then glycerine. In glycerine the mouthparts were separated from the head capsule, then the mouthparts were disarticulated. The disarticulation was transferred to melted glycerine jelly (1:1 glycerine:gelatin), positioned for optimal views of various structures, and photographed with a Nikon SMZ1500 stereoscope using Nikon Elements software.

Scanning electron microscopy (SEM) images were captured at the AMNH Microscopy and Imaging Facility using the Hitachi S-4700 FE-SEM. Prior to imaging, each specimen was mounted on a metal stub with carbon tape and sputter coated in gold palladium with a Denton Vacuum Desk V Thin Film Depositor. Images were captured at 200x magnification.

R version 4.5.1 [25] and R Studio 2025.05.0 [26] were used with the standard stats package to test for significance of difference in relative alimentary air volumes between two groups: those insects only with air in the gut vs. all examined specimens using t.test() for a two-sampled t-test. An ANOVA using aov() was also done on relative alimentary air volumes and feeding category. Categorical boxplots including significance values of the t-test results were created using ggplot2 [27].

## Results

### Inflated gut previously undocumented in several orders

**Fig 1:**
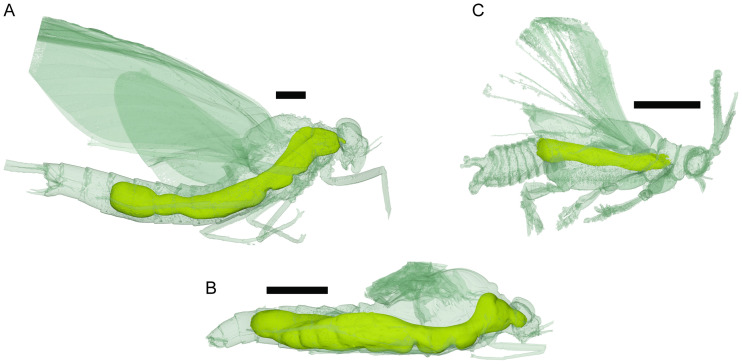
Examples of adult insects previously known to have inflated alimentary canal. Inflated alimentary canals shown in yellow. A. *Ephemera* sp. (Ephemeroptera: Ephemeridae). B. *Neocloeon triangulifer* (Ephemeroptera: Baetidae), wings removed during micro-CT scanning. C. *Xenos peckii* (Strepsiptera: Xenidae). All scale bars 1 mm.

**Fig 2:**
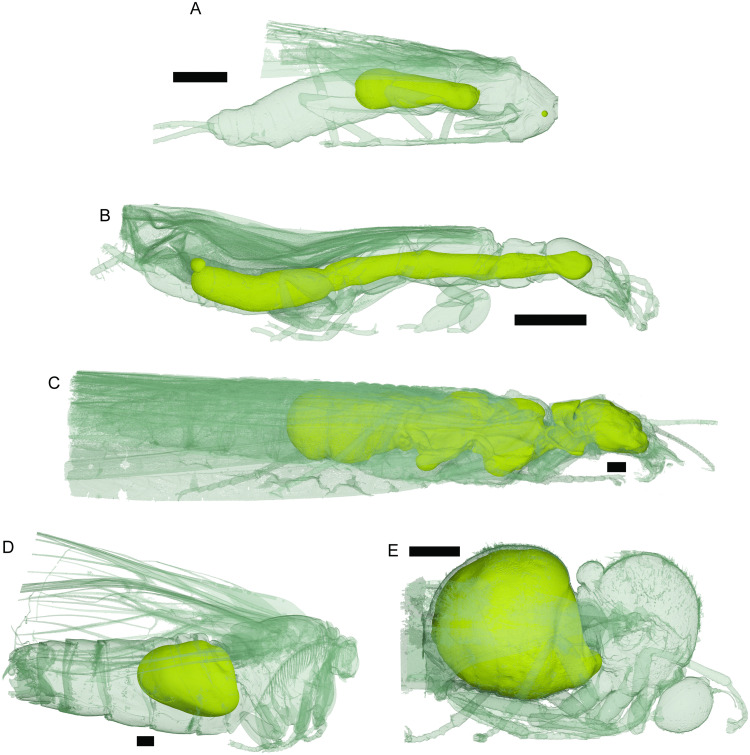
Previously undocumented inflated alimentary canals (yellow) of non-feeding adults. A. *Isoperla* sp. (Plecoptera: Perlodidae). B. *Oligotoma nigra* (Embioptera: Oligotomidae), C. *Corydalus cornutus* (Megaloptera: Corydalidae), D. *Bombyx mori* (Lepidoptera: Bombycidae), E. *Ogcodes* sp. (Diptera: Acroceridae). All scale bars 1 mm.

The inflated gut in Ephemeroptera has been known for centuries [15,16,18,28], but was only noted in Strepsiptera recently [19,20]. Using X-ray micro-CT scanning, we have imaged the inflated alimentary canal of these two orders (Fig 1), in addition to five taxa from other insect orders (Fig 2): Plecoptera (stoneflies), Embioptera (webspinners), Megaloptera (dobsonflies and alderflies), Lepidoptera (moths and butterflies), and Diptera (flies). Refer to S1 File for detailed anatomical descriptions of the inflated gut for all taxa.

#### Ephemeroptera.

Two adult mayflies, *Ephemera* sp. (Ephemeridae) and *Neocloeon triangulifer* (Baetidae), both females, were found to possess the inflated alimentary canal, a feature first noted for mayflies in general by Swammerdam [28] in the earliest microscopic dissections of insects. Both adults possess a large, elongate inflated space, beginning near (*Ephemera*) or inside (*Neocloeon*) the head capsule, proceeding through the thorax and extending through the 7^th^ abdominal segment (Fig 1A and 1B). The inflated space is constricted slightly in the thorax, likely where wing and leg muscles are positioned. An additional constriction is also present in *Ephemera* in the 4^th^ and 5^th^ segments before expansion into the final two segments of the air space; this constriction is not present in *Neocloeon*.

**Fig 3:**
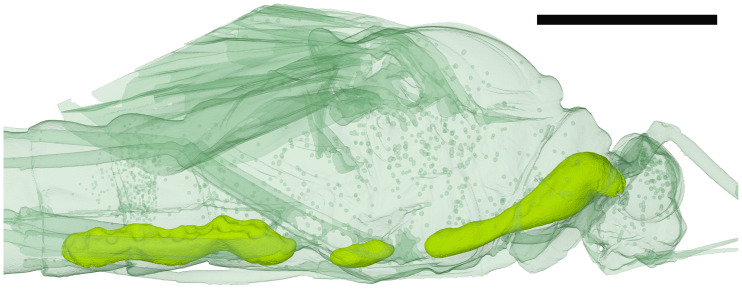
Partially filled gut in Neocloeon triangulifer (Ephemeroptera: Baetidae). Note dimpled appearance in posterior air space, possibly caused by presence of eggs. Scale bar 1 mm.

A subimago female *Neocloeon triangulifer* (Baetidae) was found to possess a partially inflated alimentary canal (Fig 3), extending in three parts from the posterior of the head capsule into the 3^rd^ abdominal segment.

#### Plecoptera.

**Fig 4:**
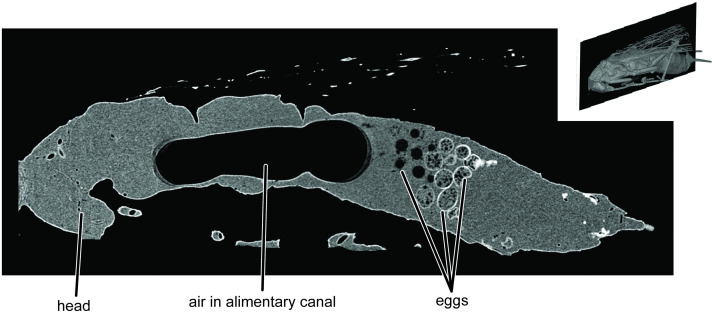
Eggs in abdomen of *Isoperla* sp. (Plecoptera: Perlodidae). Medial longitudinal X-ray section showing position of eggs in abdomen of *Isoperla*, just posterior from air space. (See inset for position of slice.).

An adult female *Isoperla* (Perlodidae) was found to have a short but pronounced inflated gut space, beginning in the mid-thorax and extending through the anterior part of the abdomen (Fig 2A). This specimen was also found to have a substantial number of eggs in various stages of development (Fig 4). We hypothesize that the inflated alimentary space in this specimen is shortened to accommodate the growth and development of the eggs.

#### Embioptera.

The adult male *Oligotoma nigra* webspinner (Oligotomidae) possesses a very long inflated cavity, beginning approximately halfway into the head capsule, just behind the eyes, and extending nearly the length of the body (Fig 2B). Several small constrictions were observed in the abdominal section of the inflated space.

#### Megaloptera.

An adult *Corydalus cornutus* (Corydalidae) female features a very large, inflated space, notably filling much of the head capsule (Fig 2C). The size of the head air space is such that it is possible the pharynx fuses with other head air sacs during development. The air space extends posteriorly through the thorax and into the abdomen, where it extends laterally almost to the inner body wall. Constrictions in the thorax are likely the result of large, paired flight muscles positioned dorsoventrally in the thorax.

#### Strepsiptera.

The adult *Xenos peckii* (Xenidae) male possesses an inflated space beginning in the second thoracic segment, extending posteriorly to the anterior portion of the abdomen (Fig 1C). Slight constrictions in the thoracic portion of the air space are likely due to the presence of flight muscles.

#### Lepidoptera.

The adult female *Bombyx mori* (Bombycidae) silkworm moth (domestic) shows a pronounced abdominal air space (which may be the crop), extending from the posterior portion of the thorax into the anterior of the 4^th^ abdominal segment (Fig 2D). The silkworm moth was the largest specimen found to have an inflated alimentary space, and while not as large relative to the rest of the body as the other insects, the known lack of adult feeding suggests possible co-opting of the digestive tract.

#### Diptera.

**Fig 5:**
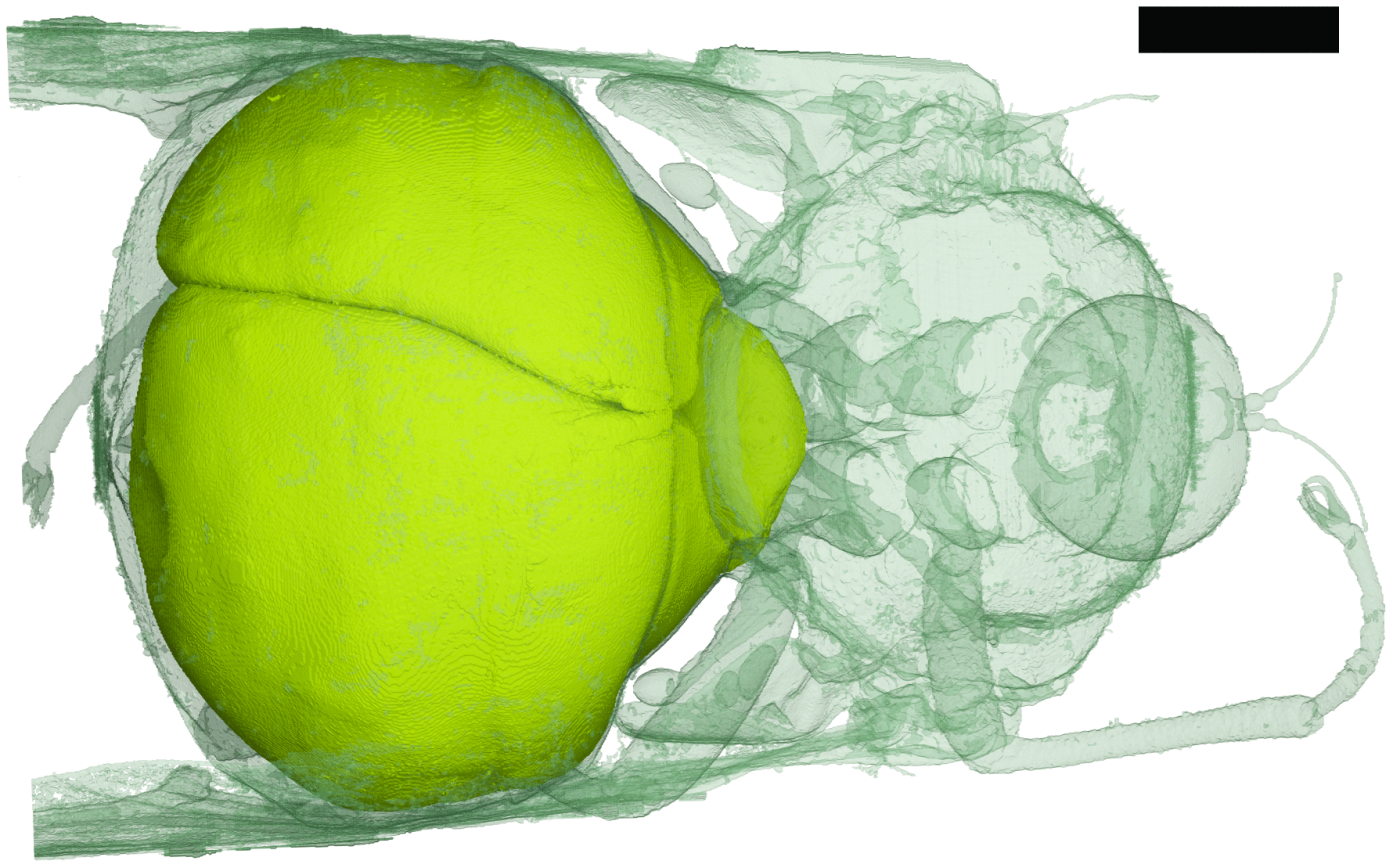
Inflated gut of spider fly *Ogcodes* sp. (Diptera: Acroceridae). Dorsal view showing bilobed nature of inflated alimentary space (including crop), filling nearly the entire abdomen. Scale bar 1 mm.

The male parasitoid spider fly *Ogcodes* sp. (Acroceridae) features an extreme example of inflated alimentary space, filling nearly the entire abdomen (Fig 1E). Indeed, the air volume comprises approximately 50% of the entire volume of the insect. The air space is bilobed (Fig 5), indicating that the crop, occasionally filled with air in Diptera during digestion [29], has been completely inflated in addition to the remainder of the alimentary canal.

### Additional evidence of adult non-feeding – mouthparts

**Fig 6:**
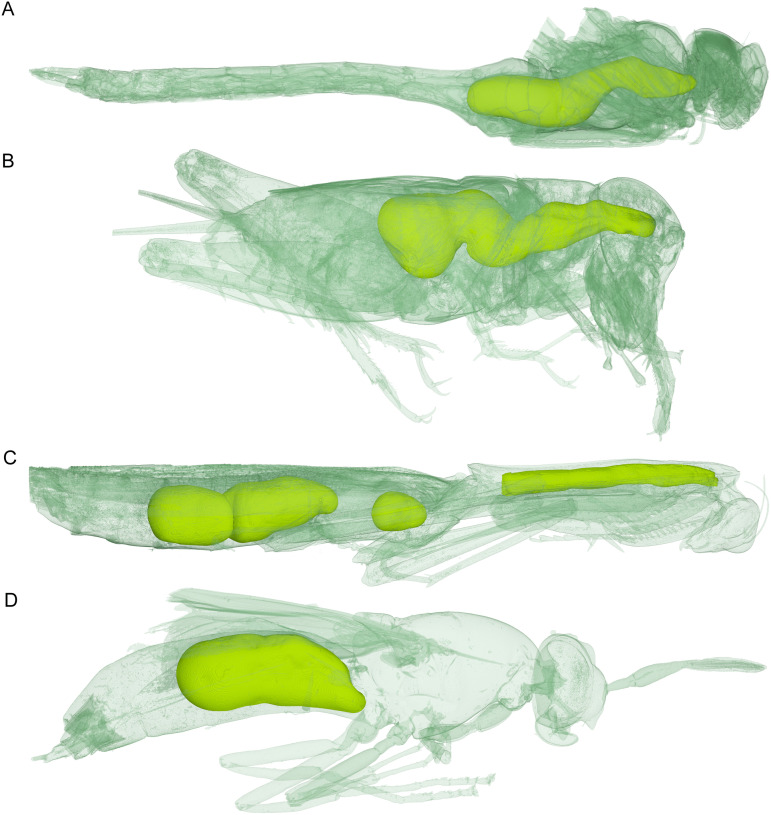
Air-filled spaces (yellow) in alimentary canal of insects known to feed as adults. A. *Anax junius* (Odonata: Aeshnidae). B. *Gryllus* sp. (Orthoptera: Gryllidae). C. *Tenodera sinensis* (Mantodea: Mantidae). D. *Hermetia illucens* (Diptera: Stratiomyidae). Insects are not on the same scale. A, B, C from [17].

An inflated insect gut is by itself insufficient evidence to prove non-feeding of the adult stage and co-opting of the alimentary canal. Even adults that feed can temporarily possess large, inflated spaces in the digestive tract [29], seen in several instances (Fig 6) in insects micro-CT scanned as part of our studies on insect respiratory morphology [17].

**Fig 7:**
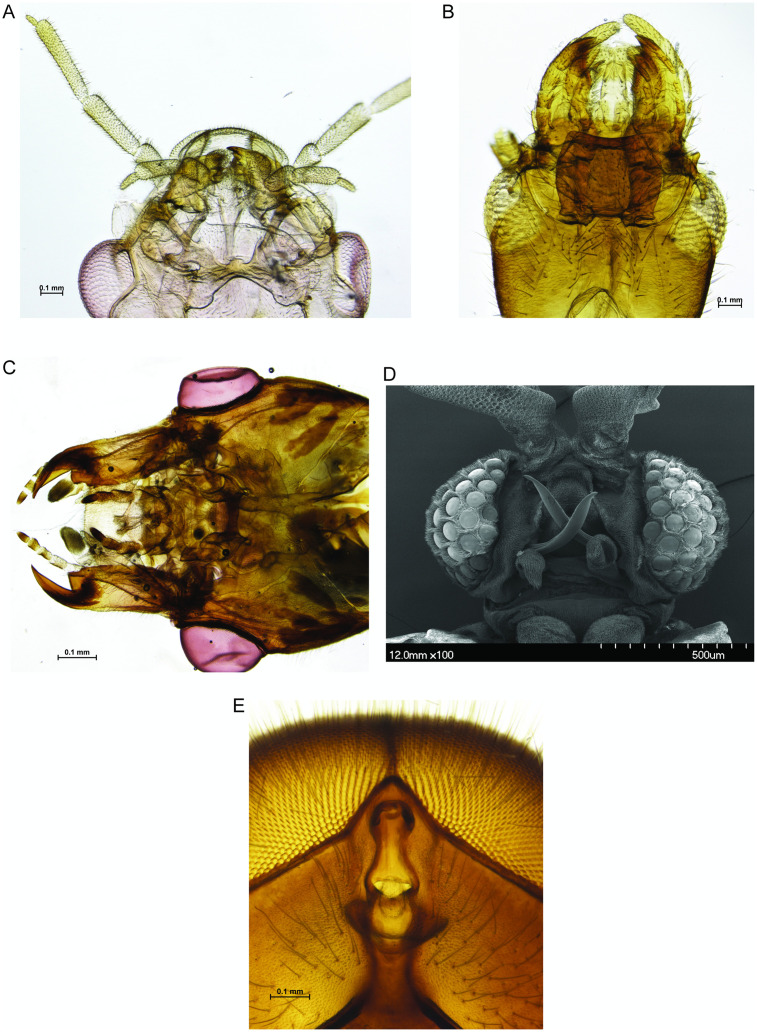
Selected heads of suspected non-feeding adults. A. *Isoperla* sp. (Plecoptera: Perlodidae). B. *Oligotoma nigra* (Embioptera: Oligotomidae), C. *Corydalus cornutus* (Megaloptera: Corydalidae), D. *Xenos peckii* (Strepsiptera: Xenidae) E. *Ogcodes* sp. (Diptera: Acroceridae). A, B, C, E photomicrographs, D Scanning-electron microscopy.

Detailed examination of adult mouthparts using dissection and light photomicrography was performed (Fig 7) to establish the likelihood of adult non-feeding and distinguish between insects with an inflated, possibly non-functional alimentary canal from those with temporary inflated spaces in the digestive tract. The *Xenos peckii* strepsipteran male shown here was imaged using scanning-electron microscopy (SEM) to expose the reduced mouthparts.

Mouthpart structure and development is one index of feeding behavior, albeit a conservative index. While insects with absent or highly vestigial mouthparts obviously do not feed, species with fully developed mouthparts may or may not feed, as we discuss for each individual taxon below. (Refer to S1 File and S1 Table for detailed anatomical descriptions of mouthparts for all taxa.)

#### Ephemeroptera.

Mayflies feed on a variety of food sources during the aquatic immature stage; while most species feed on algae and other organic matter in the water, others are carnivorous and eat other invertebrates [1]. Rather than being absent or reduced in size, adult mayflies typically possess the full complement of mouthparts, however they are weakly sclerotized and clearly non-functional for feeding [30].

#### Plecoptera.

Stonefly immatures have a varied diet; some are detritivores, feeding on decaying plant, fungal, and algal matter [31], while others are omnivorous or carnivorous. Ontogenetic changes in feeding strategy between earlier and later instars can occur in many species [32,33]. The adult female *Isoperla* specimen included here (Fig 7A) retains partially reduced mouthparts, a characteristic of the infraorder Systellognatha [33,34], which indicates that it likely does not feed on solid material.

#### Embioptera.

Web-spinners in both the immature and adult stage feed on plant material, though it is reported that adult male Embioptera do not feed [35]. They do retain the full complement of mouthparts, as with the male shown here (Fig 7B), and the mandibles are used for grasping the female during mating.

#### Megaloptera.

Megaloptera larvae are carnivorous predators, feeding on a range of small aquatic invertebrates. Adults are generally thought to not feed [36] however they may ingest small amounts of fluid, or fruit, at least in a laboratory setting [37]. In gut content analyses, no significant amounts of solid food have been detected in adult dobsonflies, and it is hypothesized that they may rely more so on fat reserves accumulated during the larval stage [36]. The female *Corydalus* included here possesses sclerotized, functional mouthparts (Fig 7C).

#### Strepsiptera.

Male Strepsiptera are known to not feed, however the *Xenos peckii* male shown here (Fig 7D) features a developed set of mandibles used for cutting open the puparium during eclosion [38]. The remaining mouthparts are highly reduced and non-functional for feeding.

#### Lepidoptera.

Larvae of *Bombyx mori*, the domesticated silkworm adult included here, are known to feed continuously with a strong preference for white mulberry. Adult *Bombyx mori* do not feed and possess highly reduced, non-functional mouthparts.

#### Diptera.

The adult *Ogcodes* acrocerid spider fly shown here possesses highly reduced mouthparts (Fig 7E). Acrocerids are also known as small-headed flies; adults in the genus *Ogcodes* have relatively short lifespans, lasting from only 3 days to up to 4 weeks, and do not feed [39]. Some acrocerid genera have long proboscides used for feeding on nectar in flowers.

### Differentiating air in gut from co-opted gut

**Fig 8:**
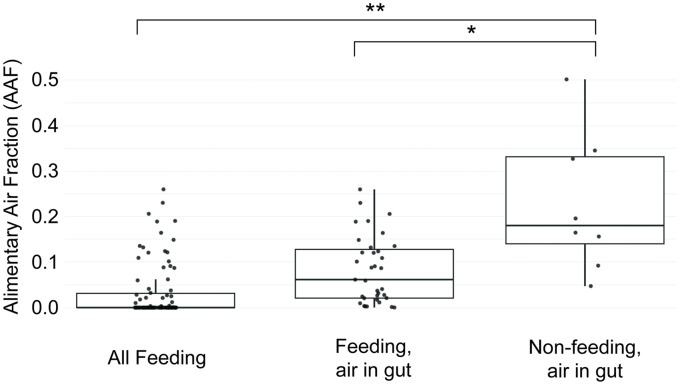
Alimentary air fraction, sorted by feeding. Insects that do not feed have disproportionately more air in the gut than those that do feed and have some air in the gut. Note many insects in the “All Feeding” category have no air in the gut. For all insects that feed compared to non-feeding insects with alimentary air, p < 0.001 (**); for insects that feed and have air in the alimentary canal compared to insects that do not feed and have air in the alimentary canal, p < 0.01 (*).

During a study of insect respiratory systems using micro-CT scanning of insect air spaces of 94 adult specimens, comprising 94 species from 25 orders (Herhold et al., in prep), 42 species were found to have some volume of air in the digestive tract across 17 orders (see Table 2). To determine if adult insects that do not feed have disproportionally larger air spaces in their digestive tract, alimentary air fraction (AAF in Table 2) is defined here as the ratio of air in the digestive tract (co-opted or otherwise) to the total volume of the insect – all air spaces including tracheae, plus integument, wings, tissue, hemolymph, etc. A two-sample t-test indicates a significant difference in the alimentary air fraction between these two groups when testing only those insects with air in the gut (n = 42, t = −2.66, p = 0.03) as well as when including all examined specimens (n = 94, t = −3.63, p < 0.01), see Fig 8. Additionally, an ANOVA with alimentary air fraction as a function of feeding type also demonstrated that non-feeding does have an impact on the alimentary air fraction for both insects with air in the gut (n = 42, F = 16.53, p < 0.001) and with all examined specimens (n = 94, F = 52.66, p < 0.001).

**Table 2:** Alimentary Air Fraction (AAF).

Division	Order	Family	Taxon	AAF
Paleoptera	Odonata	Aeshnidae	*Aeshna* sp.	0.1241
Paleoptera	Odonata	Coenagrionidae		0.0101
Paleoptera	Odonata	Libellulidae		0.0323
Paleoptera	Ephemeroptera	Baetidae	*Neocloeon triangulifer*	0.3454
Paleoptera	Ephemeroptera	Ephemeridae	*Ephemera* sp.	0.1566
Polyneoptera	Plecoptera	Perlodidae	*Isoperla* sp.	0.0927
Polyneoptera	Orthoptera	Gryllidae	*Gryllus* sp.	0.0872
Polyneoptera	Orthoptera	Tettigoniidae	*Meconema thalassinum*	0.1490
Polyneoptera	Orthoptera	Romaleidae	*Romalea microptera*	0.0413
Polyneoptera	Embioptera	Oligotomidae	*Oligotoma nigra*	0.1963
Dictyoptera	Phasmatodea	Phasmatidae	*Medauroidea extradentata*	0.0216
Dictyoptera	Phasmatodea	Phasmatidae	*Extatosoma tiaratum*	0.1325
Dictyoptera	Mantodea	Mantidae	*Tenodera sinensis*	0.1892
Dictyoptera	Mantodea	Empusidae	*Idolomantis diabolica*	0.0621
Dictyoptera	Blattodea	Blattidae	*Periplaneta americana*	0.1906
Dictyoptera	Blattodea	Blaberidae	*Blaptica dubia*	0.0283
Paraneoptera	Hemiptera	Cicadidae	*Neotibicen* sp.	0.0271
Paraneoptera	Hemiptera	Fulgoridae	*Lycorma delicatula*	0.0178
Paraneoptera	Hemiptera	Gerridae	*Aquarius remigis*	0.1094
Paraneoptera	Hemiptera	Coreidae	*Acanthocephala terminalis*	0.2062
Paraneoptera	Hemiptera	Lygaeidae	*Oncopeltus fasciatus*	0.0009
Holometabola	Megaloptera	Corydalidae	*Corydalus cornutus*	0.3272
Holometabola	Neuroptera	Chrysopidae	*Chrysoperla* sp.	0.0250
Holometabola	Strepsiptera	Xenidae	*Xenos peckii*	0.0478
Holometabola	Coleoptera	Carabidae	*Cicindela* sp.	0.0884
Holometabola	Trichoptera	Hydropsychidae		0.0914
Holometabola	Trichoptera	Limnephilidae		0.0377
Holometabola	Lepidoptera	Tineidae	*Acrolophus* sp.	0.1016
Holometabola	Lepidoptera	Sesiidae	*Synanthedon* sp.	0.1645
Holometabola	Lepidoptera	Pieridae	*Pieris* sp.	0.0599
Holometabola	Lepidoptera	Depressariidae		0.0120
Holometabola	Lepidoptera	Bombycidae	*Bombyx mori* (domestic)	0.1651
Holometabola	Lepidoptera	Sphingidae	*Manduca* sp.	0.1215
Holometabola	Mecoptera	Bittacidae	*Bittacus* sp.	0.0019
Holometabola	Diptera	Psychodidae		0.0212
Holometabola	Diptera	Rhagionidae	*Ptiolina?*	0.2304
Holometabola	Diptera	Stratiomyidae	*Hermetia illucens*	0.1357
Holometabola	Diptera	Bombyliidae	*Bombylius* sp.	0.2601
Holometabola	Diptera	Acroceridae	*Ogcodes sp.*	0.5015
Holometabola	Diptera	Asilidae	*Promachus* sp.	0.0031
Holometabola	Diptera	Asilidae		0.0041
Holometabola	Diptera	Dolichopodidae		0.0033
Holometabola	Diptera	Syrphidae	*Toxomerus* sp.	0.1208

Adult insects with alimentary canal air space, as measured by micro-CT scanning. AAF (Alimentary Air Fraction) is defined as the volume of air in the alimentary canal divided by the total volume of the insect (all enclosed air plus all tissues, cuticle, wings, etc.). The eight shaded specimens are taxa that are known or suspected to be non-feeding adults with a newly determined co-opted alimentary canal.

## Discussion

### Gas composition of inflated spaces and filling mechanisms

As the mechanism for the filling of the inflated gut has yet to be determined, the composition of the gas is not known. Harker [18] indicated that air is likely ingested by eclosing Ephemeroptera. The filling mechanisms in the remaining six orders included within this study remain unknown.

The two possibilities are either air, ingested through the mouth, or endogenous gas produced by the digestive system. Extraction and analysis of the gas would determine its origin, but the lack of digestion (producing digestive gases) in the non-feeding taxa described here suggests that these inflated spaces are filled with air. Here it will be referred to as “gas” or “air” somewhat interchangeably, with the caveat that future experimental studies are called for.

### Non-feeding adults in multiple orders

Ephemeroptera and Strepsiptera appear to be unique in that all adult mayflies and all adult (male) Strepsiptera do not feed. Adult non-feeding appears to be limited to individual species or smaller groups in all other insect orders. Here, along with Strepsiptera and Ephemeroptera, we discuss the life histories of the five other orders to establish likelihood of non-feeding and the consequent co-opting of the alimentary canal.

#### Ephemeroptera.

Mayflies have long been known to have a non-functional, inflated alimentary canal [15,16,18,28]. Combined with their exceedingly short adult lifespan and non-feeding behavior, mayflies serve as a useful exemplar in searching for other insects that may also possess a co-opted, air-filled gut.

As mentioned previously, the mechanisms for filling the inflated alimentary canal are unclear, even in mayflies, where the phenomenon has been known for some time. Connections to the tracheal system in Ephemeroptera have been proposed but not confirmed [18], and mapping of the mayfly tracheal system via micro-CT scanning indicates its isolation from the inflated gut [17]. It has been shown that rapid inflation occurs in mayfly females during oviposition, indicating that inhalation through the mouth provides air for inflation [18].

The mayfly subimago stage is unique among insects, and its developmental purpose remains unresolved (presumably a vestige of early hexapods like Zygentoma and Archaeognatha, which molt as adults) [40,41]. During this brief transitional stage of development, the insect is able to fly but is not yet sexually matured, and must molt again before becoming a reproductive adult. Subimago mayflies also do not feed, and the subimago *Neocloeon triangulifer*, micro-CT scanned as part of the previously mentioned respiratory morphology study ([17], and Herhold et al., in prep) displays a partially inflated alimentary canal (Fig 3). It is possible that reduction of digestive organs occurs in stages, whereupon the adult female is prepared to ingest air in response to oviposition. This does not resolve the question for males, however studies have suggested that ingestion of air may assist in ejaculation [18]. Pickles [15] suggested males are able to alter the degree of gut dilation, thereby changing their “specific gravity” to enable the rising and falling pattern of the mayfly “dancing flight”, however Harker [42] showed that mayflies with a constant gut volume were able to engage in swarming dance flight, indicating that rapid changes in gut air volume are not required for this behavior.

#### Strepsiptera.

All Strepsiptera males are short-lived, with a general life span of 3–6 hours, and must spend this time finding and mating with a receptive female [4]. *Xenos peckii* (Xenidae) males, for example, have been documented to live a maximum of four hours [38]; consequently, no adult males of Strepsiptera feed [3,4,20,43]. The females of Stylopidia (Strepsiptera excl. Mengenillidae), as neotenic and permanent endoparasites, have greatly reduced to vestigial mouthparts on the cephalothorax [4,44,45] and do not take in food as adults [6]. Rudimentary structures such as ommatidia, mandibles, maxilla, and labium can be seen on the female cephalothorax, but the region is primarily used for copulation and brood emergence [20,44]. The basal family Mengenillidae, sister group to all other extant taxa in the order, is the only family with free-living adult females that pupate outside of their hosts [4]. Female Mengenillidae are similar to other adult Holometabola as they do have mouthparts, however these females do not orally feed, also reliant on the host for nutrients [3,4,44].

The adult males of Strepsiptera (apart from the family Corioxenidae) generally retain moveable mandibles and maxillae [3,19,46]. The blade-shaped mandibles of *Xenos peckii*, the strepsipteran featured in this study, are used to cut open its puparium at an ecdysial suture line with a scissor-like motion, enabling the male’s emergence as an adult [38]. These blade-shaped mandibles are also present in males of the genera *Elenchus* (Elenchidae) and *Stylops* (Stylopidae) [3,4,19,47]. Use of the mandibles in breaking open the puparium has been documented for *Stylops*, but the same has not been confirmed by observation or recording for *Elenchus* [48].

In the family Corioxenidae, whose mandibles are vestigial to entirely reduced, the mode of emergence is likely a ptilinum [3] or a puparium cap that can be removed relatively easily due to a thin connection [20]. Emergence has been seen in male *Elenchus tenuicornis* via a ptilinum on the ventral side of the head between the eyes and below the antennae [49]. This differs from the positioning of the ptilinum in Diptera, but serves the same function. Kathirithamby [49] observed that male *Elenchus tenuicornis* undergo alternating inflation and deflation during the process of emergence, causing the ptilinum to repeatedly push against the pupal cap until it ruptures at a seam, very similar to the mechanism of eclosion used by teneral schizophoran flies to escape from the puparium. Though *Elenchus tenuicornis* exhibit this mode of emergence, they do still retain mandibles and maxillae [19,49]. The extent to which the ptilinum occurs in Strepsiptera is as yet unknown.

Regardless of variations in mouthpart structure and their use in eclosion, the presence of a large air space combined with adult non-feeding indicates co-opting of the alimentary canal in *Xenos peckii* and probably most or all Strepsiptera.

#### Plecoptera.

Studies on stonefly feeding have primarily focused on aquatic immatures, as these insects are essential to nutrient cycling in freshwater environments and serve as a useful bioindicator of water quality [50]. Anecdotal accounts led to the long-held assumption of widespread adult non-feeding in stoneflies [10], however this is not true for all taxa [32]. *Isoperla* (Perlodidae), a speciose Holarctic genus containing 313 species [51], has been found to feed on pollen, detritus, fungi, and algae in the imaginal stage [52,53]. The species involved in these gut studies, such as *Isoperla nevada* Aubert, 1952 or *Isoperla grammatica* Poda, 1761*,* were noted to have more developed mouthparts than other *Isoperla* [52,53]. Further, other *Isoperla* species from Great Britain and Sweden do not feed [10,54]. Thus, the feeding habits of this genus should be considered on a species-to-species basis. The volume of the air space in our female specimen (Fig 4) suggests a non-feeding adult life stage, although the presence of fully developed mouthparts is notable (Fig 7A).

Numerous species within the superfamily Perloidea feed during their adult stage [55], but the idea that adult stoneflies do not feed was perpetuated by the insistence that mouthparts are often reduced at the imaginal stage [10]. Although some Isoperlinae have somewhat reduced mouthparts, the presence of sclerotized mandibular teeth is incredibly common [32], and both adult feeding and non-feeding behaviors have been documented throughout Plecoptera [53,55–57].

As shown in Fig 4, the presence of a substantial number of eggs in the anterior portion of the stonefly abdomen may limit the size of the inflated alimentary canal. The weight of a female stonefly after egg deposition can drop by approximately 30% [58] as they take up a majority of space within an individual’s abdomen. Stoneflies generally have smaller clutch sizes than mayflies, thus depositing fewer eggs, but systellognathan stonefly eggs have a hard exterior chorion [33,34] thought to protect against environmental destruction before nymphal development (note more dense, sclerotized shells in posterior eggs; Fig 4) [59]. We hypothesize that this could limit the amount of available air space in the gut.

#### Embioptera.

Female adult webspinners differ from males in that the females have a large crop, stores of fat, and a proventriculus that aids in digestion of food [60]. Adult male webspinners lack these structures and tend to not feed after their final molt [35]. Adult male *Metoligotoma* (Australembiidae) are one of the few Embioptera that have been recorded to feed with their robust mandibles and enlarged palpi [61]. This suggests that they live longer than other male embiids, which typically die quickly after mating [62].

*Oligotoma nigra* nymphs have well-developed maxillary palps and chitinized mandibles with many denticles, indicating that they engage in biting and chewing feeding habits [63]. The mouthparts of the adult *Oligotoma* male retain much of their structure and utility, likely because webspinner males use them to grasp females during mating [35]. The thinner abdomen, as well as simpler mandibles, are both indications that adult male *Oligotoma* do not feed [63].

The inflated air space occupies the entire length of the body (Fig 2B). This non-feeding adult male specimen serves as an excellent example of a likely co-opted alimentary canal.

#### Megaloptera.

Immature life spans of the predaceous aquatic stage of megalopterans are well-known to be quite long and merovoltine, sometimes taking years to reach maturity [64]. In contrast, less is known about the reproductive, terrestrial life history of Megaloptera as the adult life spans are limited, usually only lasting between one week to one month [65,66]. It was previously thought that adult megalopterans generally do not feed, however several studies have indicated that adult females may feed on pollen, detritus, and male spermatophores to obtain substantial energy, although it is still widely accepted the females largely depend on larval energy reserves [36]. Males of *Corydalus* are unable to feed on solid food due to their large mandibles but have been observed licking the juice off of fruits [37] or honey [67] in laboratory settings, a behavior also observed in neotropical Megaloptera [36]. Adult megalopterans have been found to visit flowers for nectar or pollen [68–70], feed on tree sap [71], and consume small insects [72] in nature. As there has been much debate on whether all species of adult megalopterans feed, it is important to study the gut anatomy amongst genera and species.

#### Lepidoptera.

Across the approximately 180,000 species of the order Lepidoptera, there is considerable variation in feeding behaviors and mouthpart development. Many moth species do not feed as adults, while adult butterflies often use proboscides in nectivorous feeding [73,74]. Most notably, adults of all species in the silk moth family Saturniidae have reduced mouthparts and live abbreviated lifespans from one to two weeks, with an emphasis on reproduction and dispersal [74].

Interestingly, adults that do not feed and have reduced or atrophied mouthparts also occur in groups of Lepidoptera that are well known known to feed, including some hawk moths (Sphingidae) and ghost moths (Hepialidae). Hawk moths have a range of mouthpart development, including long proboscides for accumulating nectar [75] and host plant pollination. However, a recent study has shown that several species have lost the adult ability to feed and have reduced or nonfunctional proboscides [9].

As a model organism, the domestic silkworm *Bombyx mori* has been well known for its reduced mouthparts and non-feeding adult. The inflated alimentary space shown in the scanned adult occupies only the anterior portion of the abdomen, however it fills a substantial portion of the body cavity laterally and dorso-ventrally. In conjunction with the reduced mouthparts and known non-feeding behavior, it is likely this specimen also demonstrates a co-opted alimentary canal.

#### Diptera.

Along with Lepidoptera, Diptera is one of the four major holometabolous insect orders that, along with Hymenoptera and Coleoptera, comprise over 80% of all insect species. The approximately 170,000 known species of flies feature an extremely wide array of life histories and habitats [76]. Typical of the order Diptera, mandibles are generally lost (though retained as fine lancets or thin blades in the females of some blood-feeding groups); labial palps and glossae are fused into a pair of fleshy lobes that sponge up liquid food (in Brachycera these lobes have pseudotracheae, fine chinized tubes which assist in liquid feeding) [77–80]. Adult non-feeding is known in several groups, including several species of non-biting midges (Diptera: Chironomidae). For example, *Belgica antarctica*, a flightless midge and one of the only completely terrestrial animals native to Antarctica, spends most of its two-year lifespan in larval stages, followed by an abbreviated non-feeding adult stage.

Acroceridae is an ancient, phylogenetically isolated, small family of stout-bodied Brachycera whose larvae are parasitoids of spiders. There are dramatic differences in mouthpart development among genera, varying from highly vestigial and functionless, as in *Ogcodes*, to genera like *Eulonchus* and *Lasia*, which have extremely long proboscides used to feed on nectar and pollen from deep flowers [81,82]. Refer to S1 File for detailed descriptions of mouthpart morphology and characters.

*Ogcodes* flies are reported to not feed as adults [39]. The specimen included here features the most extreme example of an essentially completely air-filled abdomen, which appears to be divided between the crop and the alimentary canal (Figs 2E, 4). It is notable that the alimentary air space is restricted to the abdomen, possibly due to the lack of available space in the thorax, dominated by large flight muscles, and the head, which is limited in size overall.

### Air in the gut vs. co-opted gut, and convergence of the co-opted alimentary canal

As noted previously, insects that feed as adults can possess temporarily inflated spaces in the alimentary canal (Fig 6); for example, several dipteran families are known to possess a temporarily air-filled crop in-between feedings [29]. As indicated in Fig 8, non-feeding insects with an inflated gut possess disproportionately more alimentary air volume than those that do feed, supporting the hypothesis that the alimentary canal is indeed co-opted in these insects and not air-filled just by happenstance.

**Fig 9:**
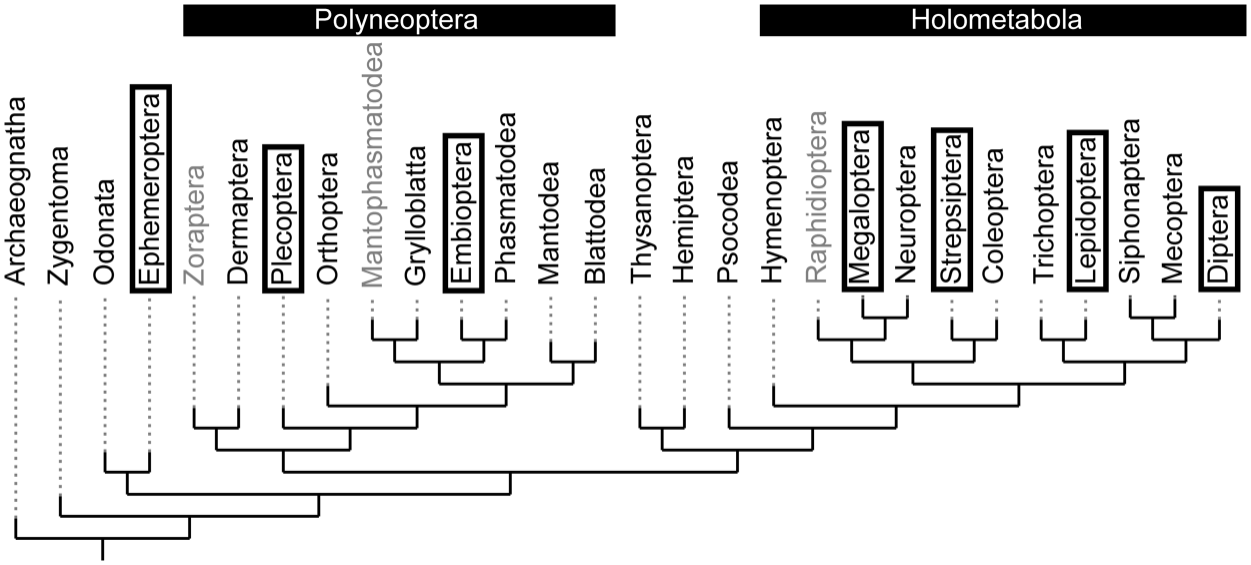
Convergence of co-opted alimentary canal among taxa studied. Phylogenetic tree of insect orders, re-drawn from [83]. Boxes indicate orders with co-opted alimentary canal that were studied here, grey text indicates unsampled orders.

As shown in Fig 9, the distribution of a co-opted alimentary canal across the higher-level divisions of class Insecta does not indicate the presence of any phylogenetic pattern or signal. The co-opted gut of Ephemeroptera, the most basal taxon to possess this trait, may indicate that the trait has been lost (or gone unused) in derived lineages. However, its sporadic appearance in both the higher-level classifications of Polyneoptera and Holometabola, for example, suggests that the co-opted inflated alimentary canal is a convergent trait.

### Possible functions of co-opted alimentary canal

Although convergence of the co-opted alimentary canal is not necessarily an indicator of adaptation, repeated and convergent development of an alimentary canal for air inflation, correlated with non-feeding in adults, indicates that the co-opted gut would not arise independently without conferring some adaptive advantage [84,85].

Its occurrence in insects with varied life histories suggests multiple possible functions, however one common, obvious trait in the adults of all species analyzed here is flight. Much of the success of insects as the dominant terrestrial lifeform can be attributed to their ability to fly. Increased dispersal rates, the ability to evade predators, and tracking of food resources are instrumental in the high speciation rates of insects. While not all insects fly, most do, and flight requires numerous physiological adaptations and high energy requirements. Flying insects that rely completely on internal energy stores feature additional adaptations. For example, the abdomens of migratory monarch butterflies contain large lipid-filled fat bodies that occupy as much as 45% of the insect’s weight [86]. Mayflies would likely be unable to fly with such adaptations, as the added mass would make flight difficult. Additionally, one of the reasons mayflies are able to disperse so far is partially because they can be driven by the wind, occasionally great distances. Hollowing out of the alimentary canal to maintain size but reduce weight, and thus energy requirements, confers a substantial advantage.

Several hypotheses have been proposed for the function of an inflated gut in mayflies, ranging from weight reduction to pneumatically assisted oviposition and ejaculation [18]. This suggests a similar purpose in stoneflies; the size of the air space and the position of the eggs in the *Isoperla* stonefly female indicates possible enlargement of the inflated gut during oviposition, though it is unclear if inflation may assist in oviposition or occurs after the fact to fill available space.

An inflated alimentary canal may serve to maintain internal hydrostatic pressure as well as body shape and volume. Reduction of the digestive tract by resorption or some other process would otherwise lead to structural collapse of the insect’s body without internal support, which is likely more important in soft-bodied insects, where this phenomenon appears to be most prevalent. Preservation of body shape and volume are probably important for weight balance in flight. Dragonfly abdomens are remarkably long, for example, but also largely air-filled [17], making the abdomen remarkably light and allowing for the center of mass of the insect to lie close to the center of lift in the thorax.

Insects known to feed as adults that have also been found to have air-filled gut spaces may also use this to maintain body volume. As shown in Fig 6, these spaces can be large, but not disproportionately so (see AAF discussion, above). While the filling mechanism remains under-studied, inspiration of air through the esophagus seems likely. Well-sclerotized insects may be less able to effectively reduce body size, so air filling of alimentary spaces during digestion may simply be dictated by fluid mechanics, as seen in the crop filling with air during digestion in tsetse [87]. Perhaps, also, gut air may serve especially to maintain body volume in adult, feeding females in which eggs are developing, as the eggs mature and require more space more gut air is lost. Mantises are a good example, as gravid females have very swollen abdomens.

Although the insect groups we identified do not consume food as adults, the persistence of an alimentary canal reflects a combination of physiological, developmental, and evolutionary factors. For instance, the alimentary canal and associated musculature may play a role in eclosion between the immature and adult stage. During eclosion, insects expand their body volume rapidly, a process driven largely by air intake, and potentially facilitated in part by the alimentary canal. The process would need to be especially rapid in species that are eclosing as aquatic nymphs, to avoid drowning (e.g., buoyancy) and predation at the water surface (and for all insects in general, before the teneral adult cuticle hardens. Regardless, these features may represent exaptations [21] as these structures no longer function for digestion, but rather non-nutritional roles. The alimentary canal’s presence may be viewed as functionally relevant in a modified physiological context in these cases.

## Conclusion and future directions

This study focuses on establishing the broad presence of a co-opted alimentary canal in adult non-feeding insects, expanding the previous census of Ephemeroptera and Strepsiptera to include 5 additional insect orders. As is so often the case, such discoveries lead to even more questions. Why do numerous insect lineages have non-feeding adults, or adults with limited capacity to feed? Non-feeding adults with short life spans could potentially be a detrimental trait, but each insect group displaying this behavior has evolved these morphological and life history attributes over thousands, often millions of years [88]. Although subjected to a short life span, the time allotted is integral for mate-finding – being able to quickly find mates leads to (although not always) non-feeding adults? If this does not always occur, then why only sometimes?

A logical next step is further studies into the functions of a co-opted gut, and given the varied life histories discussed here, it seems likely that the advantage gained by such an adaptation may be equally varied. Expansion of the gut may assist in oviposition in mayflies and stoneflies, but this is obviously not the case in male Strepsiptera or Ephemeroptera, for example.

Trait evolution can be an expensive process both in terms of time and energy. Insect wings evolved over millions of years, involving myriad pathways and accumulated advantages [89–91]. Consequently, traits often evolve for more than a single function (i.e., exaptation). Wings are critical for flight but have also been modified for protection (beetle elytra), thermoregulation (fanning honeybee hives), sound production (cricket scraper and file), inertial guidance (Diptera halteres), and probably for species recognition. Determining the mechanism and timing of inflation, mentioned previously, could shed some light on possible functions.

Another aspect that deals partially with function is the degree of co-opting and the location of the inflated space. One extreme example of this is the acrocerid fly, where the abdomen is nearly completely air-filled. The strepsipteran male, on the other hand, has a comparatively small contribution to the inflated space in the abdomen; most of the air is in the thorax. Comparatively, the thorax of the acrocerid fly has no air-filled space. Another extreme example is the *Corydalu*s female. While the abdomen contains a large part of the air-filled alimentary space, much of it lies strangely within the thorax and head, proportions which could possibly change before and after reproduction.

This study has also demonstrated the utility of micro-CT analyses for insect morphology, which has been termed a “renaissance” [22], a term with which we wholeheartedly agree. Discovery of the air-filled gut in these previously undocumented five orders occurred during a prolonged study on insect respiration and was largely accidental; indeed, given its presence in this small sampling of the one million identified insect species it seems highly likely the phenomenon is much more prevalent.

## Supporting information

S1 File Detailed Morphological Descriptions. “Telegraphic-style” morphological descriptions of inflated alimentary canal with additional views. Labeled images of mouthparts also included.
(DOCX)

S2 File Volume measurements. Body and air space volume measurements of all 94 taxa sampled for this study.
(CSV)

S1 Table Mouthpart development. Presence/absence comparative descriptions of mouthparts.
(DOCX)

S1 Fig *Ephemera* sp. (Ephemeroptera: Ephemeridae) lateral and dorsal views. (TIF)

S2 Fig *Neocloeon triangulifer* (Ephemeroptera: Baetidae) adult. (TIF)

S3 Fig *Neocloeon triangulifer* (Ephemeroptera: Baetidae) sub-imago. (TIF)

S4 Fig *Isoperla* sp. (Plecoptera: Perlodidae) adult. (TIF)

S5 Fig *Isoperla* sp. (Plecoptera: Perlodidae) mouthparts, ventral view. (TIF)

S6 Fig *Oligotoma nigra* (Embioptera: Oligotomidae) male adult. (TIF)

S7 Fig *Oligotoma nigra* (Embioptera: Oligotomidae) mouthparts, ventral view. (TIF)

S8 Fig *Corydalus cornutus* (Megaloptera: Corydalidae) male adult. (TIF)

S9 Fig *Corydalus cornutus* (Megaloptera: Corydalidae) mouthparts, ventral view. (TIF)

S10 Fig *Xenos peckii* (Strepsiptera: Xenidae) adult male. (TIF)

S11 Fig *Xenos peckii* (Strepsiptera: Xenidae) mouthparts. (TIF)

S12 Fig *Bombyx mori* (Lepidoptera: Bombycidae) alimentary air space. (TIF)

S13 Fig *Ogcodes* sp. (Diptera: Acroceridae) adult lateral (top) and dorsal (bottom) views. (TIF)

S14 Fig *Ogcodes* sp. (Diptera: Acroceridae) mouthparts, ventral view. (TIF)
