# Supplementary material for: Convergence of alimentary air inflation and adult non-feeding in insects, and possible adaptive functions
Source: PLoS One. 2026 Jun 11;21(6):e0351543. doi: 10.1371/journal.pone.0351543 (PMC13258001; doi:10.1371/journal.pone.0351543)
Supplement: S1 File — “Telegraphic-style” morphological descriptions of inflated alimentary canal with additional views. Labeled images of mouthparts also included. (DOCX) [file pone.0351543.s001.docx]

S1 File, Supporting Information for Herhold, Davis, Millena, Eichert, Markee, and Grimaldi, “Convergent evolution of inflated alimentary canal in non-feeding adult insects”.

# Detailed Morphological Descriptions

## Ephemeroptera

Micro-CT Scanning: *Ephemera* sp. (Ephemeroptera: Ephemeridae) and *Neocloeon triangulifer* (Ephemeroptera: Baetidae) adults with large inflated space, beginning in head and proceeding through thorax and abdomen, extending through 7^th^ abdominal segment (S1 Fig, *Ephemera,* and S2 Fig, *Neocloeon*). Anterior end of *Neocloeon* air space extending into head capsule, in *Ephemera* beginning just posterior from eyes. Abdominal longitudinal tracheae compressed laterally up against body wall by inflated alimentary air space, especially in *Neocloeon* adult. *Neocloeon* sub-imago specimen (S3 Fig) features incompletely filled alimentary canal, divided into three sections that fuse into a single space in adult; first section extending into head capsule, similar to adult, yet ending halfway between meta- and meso-thoracic spiracles; second section, smallest of the three, located ventrally from metathoracic spiracle; third section beginning at first abdominal section and extending posteriad through third abdominal segment.

S1 Fig. *Ephemera* sp. (Ephemeroptera: Ephemeridae) lateral and dorsal views. Inflated gut shown in yellow.

S2 Fig. *Neocloeon triangulifer* (Ephemeroptera: Baetidae) adult.

S3 Fig. *Neocloeon triangulifer* (Ephemeroptera: Baetidae) sub-imago.

Mouthparts: Ephemeroptera mouthparts very membranous and non-functional for adult feeding. Clypeus absent. Mandibles present but membranous and non-functional. Maxillary mouthparts small and fused; maxillary palps 3-merous. Labium present, with postmentum, prementum, glossa, paraglossa, and labial palps all completely membranous. Labial palps 3-merous. Oral cavity as a small, anteroventral pore, with cibarium absent. Pharynx and tentorum present, with the latter poorly sclerotized.

## Plecoptera

Micro-CT Scanning: *Isoperla* sp. female, with large air space beginning in thorax between meso- and meta-thoracic spiracles, extending posteriad into second or third abdominal segment (see S4 Fig). Abdomen with many eggs in various stages of development (see Fig 3 in main text); presence of eggs may limit air space size. Small air bubble in head capsule; possibly in alimentary canal (or preservational artifact).

S4 Fig. *Isoperla* sp. (Plecoptera: Perlodidae) adult.

Mouthparts: (S5 Fig.) Clypeus present and well-developed. Mandibles well-developed for chewing, with sclerotized teeth present. Labrum present. Maxillary mouthparts present, with cardo, stipes, galea, lacinia, and maxillary palps all well-developed. Lacinia with inner spicules, maxillary palps 4-merous. Labium likewise, with postmentum, prementum, and glossa present; paraglossa and labial palps well developed with palps 3-merous. Oral cavity and cibarium present, with pharynx present and funnel-shaped. Tentorium well-developed.

S5 Fig. *Isoperla* mouthparts, ventral view.

## Embioptera

Micro-CT Scanning: *Oligotoma* male (S6 Fig) air space beginning in head capsule and proceeding posteriad, nearly length of the body, terminating almost at end of abdomen. Similar to *Neocloeon* mayfly (Ephemeroptera), abdominal portion of alimentary canal greatly inflated, pressing longitudinal tracheae up against inner body wall.

S6 Fig. *Oligotoma nigra* (Embioptera: Oligotomidae) male adult.

Mouthparts: (S7 Fig.) Clypeus, labrum, and mandibles well-developed, with large, dentate, sclerotized teeth for chewing. Maxillary mouthparts present, with well-developed cardo and stipes. Galea present. Lacinia well-developed with apical teeth and inner spicules, maxillary palps well-developed and 5-merous. Labium with postmentum well-developed; prementum present, small glossa; paraglossa and labial palps well-developed with palps 3-merous. Oral cavity and cibarium present. Pharynx also present but very narrow. Tentorium present.

S7 Fig. *Oligotoma nigra* mouthparts, ventral view. Note that the tentorium is not very well developed.

## Megaloptera

Micro-CT Scanning: Female *Corydalus cornutus*, S8 Fig. Alimentary air space large and broad, beginning in head capsule, even with eyes and extending posteriad through third abdominal segment. Head portion extending dorsally and laterally nearly up against head capsule wall; head tracheae primarily ventral. Thoracic portion similar, pressing against dorsal longitudinal trunks and extending into coxae. Abdominal section greatly inflated, pushing tracheae up against inner body wall.

S8 Fig. *Corydalus cornutus* (Megaloptera: Corydalidae) male adult.

Mouthparts: (S9 Fig.) Clypeus, mandibles, and labrum well-developed. Mandibles large, dentate with sclerotized teeth. Maxillary mouthparts present, with well-developed cardo, stipes, and brushy galea. Lacinia setose and not toothed. Maxillary palps well-developed and 5-merous. Labium with well-developed postmentum; prementum present with small, setose glossa. Paraglossa present, labial palps well-developed and 3-merous. Oral cavity present, cibarium not visible. Pharynx present but very narrow and possibly incomplete, Tentorium present. Large tracheal bullae visible.

S9 Fig. *Corydalus* mouthparts, ventral view.

## Strepsiptera

Micro-CT Scanning: Abdominal air space of *Xenos* sp., a male (S10 Fig), beginning anterior of mesothoracic spiracle and extending through thorax, ending in first abdominal segment.

S10 Fig. *Xenos* sp. (Strepsiptera: Xenidae) adult male.

Mouthparts: (S11 Fig.) Mouthparts highly reduced. Clypeus absent; mandibles present but apically blade-like and adapted for cutting open puparium[1-3] (see S11 Fig). Maxillary mouthparts absent with exception of small maxillary palp, 1-merous. Labial mouthparts with post- and pre-mentum fused, just beneath small oral opening. Glossa, paraglossa, and labial palps absent. Oral cavity as small, anterior pore. Cibarium, pharynx, and tentorium all absent.

S11 Fig. *Xenos* sp. (Strepsiptera: Xenidae) mouthparts. (A) photomicrograph of cleared specimen, (B) SEM of mouthparts of another specimen of same species; note blade-like mandibles [1].

## Lepidoptera

Micro-CT Scanning: *Bombyx mori* alimentary air space restricted to but nearly filling anterior half of abdomen. Posterior half of abdomen highly tracheated.

S12 Fig. *Bombyx mori* alimentary air space.

Mouthparts: Mandibles absent (as in 99% of all Lepidoptera, the Glossata); galeae (the feeding structures for Glossata) modified into paired, bladder-like structures that do not meet in middle (obviously not functional for feeding, unlike the pair of “zippered” galeal valves that form a siphonate tube [proboscis] of Lepidoptera adults that feed); maxillary palpi absent, labial palpi small, short, 1-segmented; tentorium fairly well developed, anterior end surrounding interior base of antennae; cibarium small, poorly sclerotized, vestigial.

## Diptera

Micro-CT Scanning: Acrocerid alimentary air space (S13 Fig), a large, bilobed structure nearly completely filling abdomen but absent from thorax and head.

S13 Fig. *Ogcodes* sp. (Diptera: Acroceridae) adult lateral (top) and dorsal (bottom) views.

Mouthparts: Acroceridae (S14 Fig): *Ogcodes* sp. Mouthparts highly vestigial; mouth opening very small, clypeus a minute u-shaped sclerite anterior to this; labellum present as a minute, membranous button lacking pseudotracheae; maxillar palps and other maxillary appendages absent, tentorial arms absent; an internal, scoop-shaped sclerite present (probably a remnant of cibarium, Peterson [4] interprets this as the hypopharynx), one end adjacent to oral opening. There is no trace of an esophagus or pharynx.

S14 Fig. *Ogcodes* sp. (Diptera: Acroceridae) mouthparts, ventral view.

# References

1. Hrabar M, Danci A, McCann S, Schaefer PW, Gries G. New findings on life history traits of *Xenos peckii* (Strepsiptera: Xenidae). The Canadian Entomologist. 2014;146(5):514–27.

2. Kathirithamby J. Review of the Order Strepsiptera. Systematic Entomology. 1989;14(1):41–92.

3. Beutel RG, Pohl H. Head structures of males of Strepsiptera (Hexapoda) with emphasis on basal splitting events within the order. J Morphol. 2006;267(5):536–54.

4. Peterson A. The head-capsule and mouth-parts of Diptera. Illinois Biological Monographs. 1916;3(112).
