# Supplementary material for: Convergence of alimentary air inflation and adult non-feeding in insects, and possible adaptive functions
Source: PLoS One. 2026 Jun 11;21(6):e0351543. doi: 10.1371/journal.pone.0351543 (PMC13258001; doi:10.1371/journal.pone.0351543)
Supplement: S1 Table — Presence/absence comparative descriptions of mouthparts. (DOCX) [file pone.0351543.s003.docx]

S1 Table. Mouthpart development.

|  | Ephemeroptera  Two spp. | Plecoptera  (Perlodidae) | Embioptera  *Oligotoma* | Megaloptera  *Corydalus* | Strepsiptera  *Xenos* | Lepidoptera  *Bombyx* | Diptera  *Ogcodes* |
| --- | --- | --- | --- | --- | --- | --- | --- |
| Clypeus | – | ++  Well-developed | ++ | ++ | – | +  (small) | –  (minute? Internal) |
| Mandibles | +  (membranous) | ++ | ++ | ++ | +  (not chewing, apically blade-like) | –  (absent, as in all Glossata) | –  (lost: Diptera) |
| Labrum | ? | ++ | ++ | ++ | – | – | – |
| Maxilla: |  |  |  |  |  |  |  |
| Cardo | Small, ?Fused  (completely membranous) | ++ | ++ | ++ | – | – | – |
| Stipes | Small, ?Fused  (completely membranous) | ++ | ++ | ++ | – | – | – |
| Galea | +  (minute, completely membranous) | ++ | + | ++ | – | +  (bladder-like, non-functional) | –  (lost: Diptera) |
| Lacinia | –  (completely membranous) | ++ | ++ | + | – | – | –  (lost: Diptera) |
| Max palp | +  3-merous  (completely membranous) | ++  4-merous | ++  5-merous | ++ | +  1-merous | – | –  (lost: Diptera) |
| Labium: |  |  |  |  |  |  |  |
| Postmentum | +  (completely membranous) | + | ++ | ++ | Fused, just beneath oral “pore” | – | – |
| Prementum | +  (completely membranous) | + | + | + | Fused, just beneath oral “pore” | – | – |
| Glossa | +  (completely membranous) | +  w/ inner spicules | +  Small | + | – | – | Labellum minute button, no pseudotracheae |
| Paraglossa | +  (completely membranous) | ++ | ++ | + | – | – | Labellum minute button, no pseudotracheae |
| Lab. Palp | +  3-merous  (completely membranous) | ++  3-merous | ++  3-merous | ++  3-merous | – | +  (v. small, 1-merous) | Labellum minute button, no pseudotracheae |
| Oral cavity | +  (small, anteroventral pore) | + | + | + | +  (small, anterior pore) | +  (small) | +  (minute pore) |
| Cibarium | – | + | + | ?– | – | +  (small, weak) | +?  (inner scoop-shaped sclerite) |
| Pharynx | + | +  Funnel-shaped | +  Very narrow | + | – | – | – |
| Tentorium | +  (poorly sclerotized) | ++ | + | + | – | + | – |
| Feeds? | No | Limited? Behavior: no? | Behavior:  No | Behavior:  Limited?  (fluids?) | No | No | No |
| Notes |  |  |  | Large paired tracheal “bullae” in head |  |  |  |
